# Spectrum of Osteoporosis Etiologies with Associated Vertebral Compression Fractures in Children: Analysis of 11 Cases

**DOI:** 10.3390/jcm15010123

**Published:** 2025-12-24

**Authors:** Sara Aszkiełowicz, Anna Łupińska, Izabela Michałus, Arkadiusz Zygmunt, Renata Stawerska

**Affiliations:** 1 Department of Endocrinology and Metabolic Diseases, Polish Mother’s Memorial Hospital—Research Institute, 93-338 Lodz, Poland; sara.aszkielowicz@iczmp.edu.pl (S.A.); izabela.michalus@iczmp.edu.pl (I.M.); renata.stawerska@umed.lodz.pl (R.S.); 2 Department of Developmental Age and Adult Endocrinology, Medical University of Lodz, 93-338 Lodz, Poland

**Keywords:** vertebral fractures, pediatric osteoporosis, fractures in children

## Abstract

**Background/Objectives**: Pediatric osteoporosis is a multifactorial condition characterized by impaired bone mineralization and increased fracture risk, particularly vertebral compression fractures. This study aims to evaluate the diverse etiology, diagnostic challenges, and treatment options for pediatric osteoporosis in a cohort of affected children. **Methods**: We reviewed eleven pediatric patients (aged 5–16 years) diagnosed with vertebral fractures and osteoporosis, who were hospitalized between 2020 and 2024 at the Department of Endocrinology and Metabolic Diseases at PMMH-RI in Lodz. Clinical evaluation included medical history, physical examination, biochemical markers of bone metabolism, and imaging techniques such as dual-energy X-ray absorptiometry (DXA) to determine underlying causes of bone fragility. **Results**: The cohort presented a broad etiological spectrum, including seven patients with genetic disorders (e.g., mutations in *COL1A1*, *LRP5*, *SGMS2*, and *ALPL* genes) and secondary osteoporosis due to chronic diseases requiring prolonged glucocorticoid therapy (two patients with Duchenne muscular dystrophy (DMD), one patient with Crohn’s disease) or endocrinological disorders (one patient with Cushing disease). Vertebral fractures were confirmed in all patients, with back pain as the predominant symptom. Low bone mass (BMD Z-score < −2.0) was observed in eight individuals; in others, clinical signs of skeletal fragility were present despite Z-scores above this threshold. Mild biochemical abnormalities included hypercalciuria (3/11 cases) and vitamin D deficiency (6/11 cases). Height adjustment improved BMD interpretation in short-stature patients. Most children received bisphosphonate therapy, supplemented with calcium and vitamin D. In two patients, bisphosphonates were not used due to lack of parental consent or underlying conditions in which such treatment is not recommended. **Conclusions**: Pediatric osteoporosis requires a multidisciplinary diagnostic and therapeutic approach, integrating clinical, biochemical, and genetic factors. It is a heterogeneous and often underrecognized condition, with vertebral fractures frequently serving as its earliest sign—even in the absence of overt symptoms or low bone mass. This underscores the need for clinical vigilance, as significant skeletal fragility may occur despite normal BMD values. Importantly, pediatric osteoporosis may also impact the attainment of peak bone mass and ultimately affect final adult height. Early diagnosis through thorough assessment, including height-adjusted DXA, and a multidisciplinary approach are essential to ensure timely management and prevent long-term complications.

## 1. Introduction

Pediatric osteoporosis represents a growing and multifaceted concern within the field of pediatrics, characterized by a significant disruption in bone growth and mineralization processes that are critical to a child’s physical development. According to the International Society for Clinical Densitometry (ISCD), the diagnosis of osteoporosis in children and adolescents should not be based solely on densitometric criteria. The presence of one or more vertebral compression fractures suggests osteoporosis, provided there is no local disease or high-energy trauma involved. If vertebral compression fractures are absent, osteoporosis can be diagnosed with a combination of a clinically significant fracture history and a Bone Mineral Density (BMD) Z-score ≤ −2.0. A clinically significant fracture history includes the following: (1) two or more long bone fractures by age 10; or (2) three or more long bone fractures at any age up to 19 years. A Bone Mineral Content (BMC) or BMD Z-score above −2.0 does not rule out the possibility of skeletal fragility or an increased risk of fractures [1]. In recent years, the diagnosis of osteoporosis in children has increasingly focused on clinical context and fracture history rather than BMD—ISCD emphasizes that the diagnosis must integrate not only densitometric but also clinical findings to account for the dynamic nature of skeletal growth during childhood and adolescence [1].

The etiology of pediatric osteoporosis is diverse and complex, encompassing primary and secondary causes [2]. Primary osteoporosis, as seen in genetic disorders like OI, results from intrinsic abnormalities in bone matrix production or mineralization—these defects can affect collagen synthesis, bone mineralization, or the function of osteoblasts and osteocytes. Secondary osteoporosis arises from external factors that disrupt the delicate balance of bone turnover, a process characterized by the interplay of bone resorption by osteoclasts and bone formation by osteoblasts. This balance is particularly vital during periods of accelerated skeletal growth, such as infancy, childhood, and adolescence. Conditions such as chronic inflammatory diseases (e.g., juvenile idiopathic arthritis or inflammatory bowel disease), endocrine disorders (e.g., hypogonadism, hypercortisolism), malnutrition, and prolonged exposure to glucocorticoid therapy are among the prominent contributors to secondary osteoporosis in children [3].

Because pediatric osteoporosis is rare, population-level incidence data are not well established. However, among primary forms, OI represents the most common type of genetically determined bone fragility and, by definition, fulfills the criteria for osteoporosis, with an estimated incidence of 1 in 15,000 to 20,000 births [4]. In contrast, secondary osteoporosis has been reported in up to 39% of children with Crohn’s disease [5] and up to 60% of those with Duchenne muscular dystrophy (DMD) [6].

Although less common, pediatric osteoporosis has important clinical consequences; vertebral fractures may occur without significant trauma and can be the earliest indication of a systemic skeletal condition [7]. These fractures may be associated with a spectrum of complications, including chronic pain, permanent vertebral deformities and impairments in musculoskeletal function [8], all of which can severely impact a child’s quality of life, growth trajectory, and functional independence.

The clinical management of pediatric osteoporosis necessitates an individualized and multidisciplinary approach. Pharmacologic anti-resportive treatments, such as use of bisphosphonates, have shown promise in increasing bone density and reducing fracture risk in pediatric populations [9,10]. However, their long-term safety profile and effects on the growing skeleton remain areas of active investigation. As an alternative pharmacologic intervention, supplementation with calcium and vitamin D plays a crucial role in supporting bone health. Additionally, promoting regular physical activities is critical for enhancing bone strength and overall musculoskeletal health.

In light of these complexities, this study aims to highlight pediatric osteoporosis as a clinically significant yet often underrecognized condition, particularly through the lens of vertebral fractures as its hallmark manifestation. By focusing on vertebral fractures as a window into the broader pathophysiology of pediatric osteoporosis, the goal is to emphasize the need for early detection and individualized management

## 2. Materials and Methods

### 2.1. Patients Included in the Study

This report details eleven pediatric cases—five female and six male patients—aged between 5 and 16 years, who were hospitalized between 2020 and 2024 at the Department of Endocrinology and Metabolic Diseases at PMMH-RI in Lodz. The study population consisted of pediatric patients aged 5–18 years who met the inclusion criteria of a primary or secondary diagnosis of osteoporosis with an established and clearly identified etiology, accompanied by low-energy vertebral compression fractures. All patients had a well-documented clinical background and complete medical records. Written informed consent was obtained from parents or legal guardians prior to enrollment. Patients were excluded if vertebral fractures resulted from high-energy trauma or other causes unrelated to underlying bone fragility, in order to focus specifically on osteoporosis-related fragility fractures. Additional exclusion criteria included age below 5 years and lack of parental or legal guardian consent. The etiology and clinical presentation of these disorders were highly varied, reflecting a complex interplay between genetic, disease-related, and treatment-related factors. Within the cohort, the etiological spectrum for the bone mineralization disorders was diverse, as shown in Table 1. Based on a thorough review of patients’ medical records, genetic testing results were available for eight individuals. Approval for the study was obtained from the Bioethical Committee at the Polish Mother’s Memorial Hospital Research Institute (PMMH-RI) in Lodz, Poland.

Two patients (Patient 1, Patient 2) presented with DMD, a progressive genetic disorder characterized by muscle degeneration due to mutations in the DMD gene. DMD patients are predisposed to bone density reduction and compression fractures due to decreased mobility and the frequent requirement of corticosteroid therapy, which contributes to bone demineralization.

Patient 3 had a history of Crohn’s disease and presented with secondary osteoporosis, likely attributable to prolonged corticosteroid therapy, a standard but high-risk treatment for managing autoimmune inflammation [11]. Corticosteroids are well-documented to inhibit bone formation and enhance bone resorption, significantly increasing fracture risk. Additionally, the underlying disease itself, as a malabsorptive disorder, may further impair bone mineralization by reducing the absorption of essential nutrients, exacerbating skeletal fragility [12,13].

Patient 4 harbored a mutation in the Collagen, type I, alpha 1 (*COL1A1*) gene, which encodes type I collagen, a fundamental structural protein of the bone matrix. Other two patients—Patient 6 and Patient 11—had a mutation in Collagen, type I, alpha 2 (*COL1A2*), a gene similarly involved in collagen synthesis. Mutations in *COL1A1* and *COL1A2* are implicated in collagenopathies, including osteogenesis imperfecta, a disorder characterized by increased bone fragility and a high predisposition to fractures [14].

Patient 5 carried mutations in the Low-density lipoprotein receptor-related protein 5 (*LRP5*) gene, a key regulator of bone mass through its role in the Wnt signaling pathway. Pathogenic variants in *LRP5* are linked to osteoporosis-pseudoglioma syndrome, a disorder characterized by markedly reduced bone density and a heightened fracture risk [15].

The remaining two patients (Patient 7, Patient 8) exhibited mutations in the Sphingomyelin synthase 2 (*SGMS2*) gene, which has been increasingly associated with impaired bone mineralization and skeletal fragility. While the exact pathogenic mechanisms underlying SGMS2-related bone disease remain under investigation, emerging evidence suggests its involvement in bone strength regulation and fracture susceptibility [16].

Patient 9, who had Cushing’s disease secondary to a pituitary adenoma, was unusual in that he did not present with the obesity typically observed in pediatric cases; instead, the predominant clinical feature was growth retardation, first noted around 11 years of age and back pain. Genetic testing for common and rare variants associated with pituitary adenomas (including *MEN1*, *AIP*, and *USP8* mutations) did not reveal any pathogenic variants.

Additionally, Patient 10 was found to have a mutation in the *ALPL* gene, which encodes tissue-nonspecific alkaline phosphatase (TNSALP), a crucial enzyme involved in bone mineralization. ALPL mutations are responsible for hypophosphatasia, a metabolic bone disorder characterized by defective skeletal mineralization, low serum alkaline phosphatase activity, and an increased risk of fractures [17,18].

### 2.2. Clinical Examination

In all children, a comprehensive medical history was obtained, encompassing detailed information on primary symptoms including pain complaints, fracture history, comorbid conditions, and current pharmacological treatments. During the physical examination, parameters such as height, body weight, and pubertal development according to Tanner stage were meticulously assessed.

### 2.3. Biochemical Parameters

All patients underwent an in-depth evaluation of calcium-phosphate metabolism parameters, which included serum levels of calcium, phosphate, parathyroid hormone (PTH), and 25-hydroxyvitamin D (25-OHD), as well as the activity of alkaline phosphatase. 25-OHD levels between 30 and 50 ng/mL were considered within the optimal reference range, consistent with guidelines suggesting this interval supports musculoskeletal health and adequate bone mineralization [19,20]. Additionally, bone turnover markers were assessed, including serum osteocalcin and β-CrossLaps (C-terminal telopeptide of type I collagen), to provide a more comprehensive view of bone remodeling dynamics. The calcium-to-creatinine ratio in a urine sample was also measured to assess renal calcium excretion, offering valuable insight into calcium metabolism and potential dysregulation. All laboratory analyses were performed at the same PMMH-RI facility in Łódź, following standardized protocols. Serum concentrations of osteocalcin and CrossLaps were determined using the electrochemiluminescence immunoassay (ECLIA) method on a Roche Cobas automated analyzer.

### 2.4. Imaging Techniques

A radiographic evaluation of the spine was performed in all patients to assess vertebral integrity, using lateral thoracic and lumbar spine radiographs to analyze the height and morphology of the vertebral bodies, in accordance with standard diagnostic protocols [21]. For five patients, where clinical suspicion of fractures in other anatomical regions was high, additional imaging studies were performed to evaluate these areas comprehensively. Furthermore, BMD was assessed in all patients using DXA with Hologic Horizon Bone Densitometry System (MAN04871-3402 version 007). For each patient, DXA assessments were performed at two sites: Total Body Less Head (TBLH) and the Lumbar Spine (Spine). Measurements included both the TBLH and Spine projections, enabling the calculation of Z-scores.

In patients with short stature (hSDS score below -2), BMD was adjusted using the Height-Adjusted Z-Score (HAZ), allowing for more accurate interpretation relative to their height percentiles. The adjusted Z-scores were calculated according to the formula [22]:

BMDHAZZ-score = BMDageZ-score − HAZ-predicted BMD Z-score

These standardized scores facilitated the evaluation of bone density relative to age-matched norms, body size, providing insights into bone strength and fracture risk.

## 3. Results

Anthropometric measurements, clinical features, and the stage of pubertal development were collected for all patients. Data include sex, age, Tanner stage, height with corresponding height standard deviation scores (hSDS), presence of short stature, weight, BMI with percentile ranges, initial clinical symptoms, and underlying diagnoses. These characteristics are summarized in Table 1, providing a comprehensive overview of the variability in growth, pubertal development, and clinical presentation among patients.

Biochemical assessments—including serum calcium, phosphate, parathyroid hormone, and alkaline phosphatase—did not reveal major disturbances in calcium-phosphate metabolism in most patients. However, mildly increased urinary calcium excretion was observed in three individuals (patients 5, 9, and 10). Vitamin D deficiency (25(OH)D < 30 ng/mL) was detected in three patients (patients 7, 10, and 11) necessitating supplementation (Table 2).

**Figure 1:**
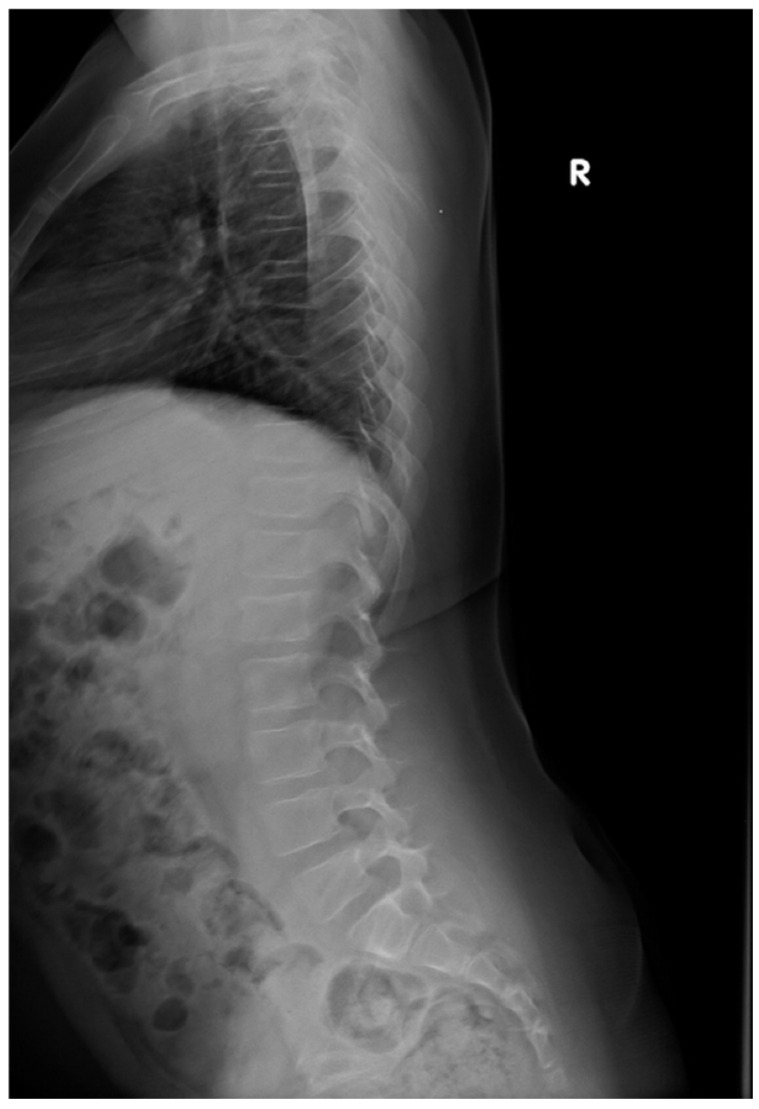
X-ray image of Patient 1 showing fractures in the vertebrae Th7, Th11, and L1.

**Figure 2:**
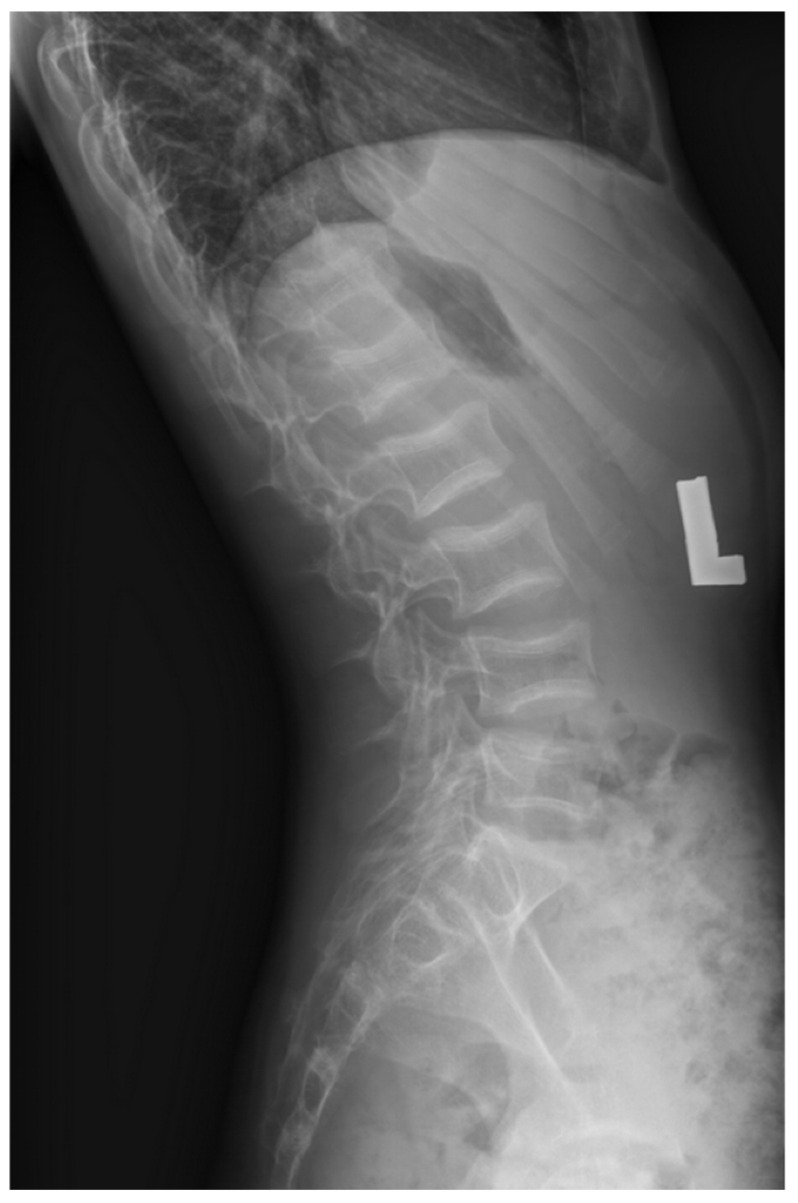
X-ray image of Patient 11 showing fractures in the vertebrae Th11-L5.

The initial diagnosis of vertebral compression fractures was confirmed through spine X-ray imaging in multiple projections (Figure 1 and Figure 2). In six children, the medical history indicated fractures of additional bones (Table 3). Out of the 11 patients included in the study, 6 individuals had fractures outside of the vertebral column. These extra-vertebral fractures were predominantly located in the forearm (patients 1, 5), femur (patient 6), tibia (patient 4), humerus (patient 11), sternum (patient 3), and shoulder (patient 6). In all patients except for patient 4, fractures involved multiple vertebrae across both the thoracic, lumbar or even cervical regions. Patient 4, however, had a fracture limited to a single vertebra, L1.

Low bone mass, defined as a BMD Z-score below −2.0, was observed in the majority of patients. Two individuals (patients 5 and 10) did not meet the densitometric criterion for low bone mass, despite clinical features suggestive of impaired bone health. Specifically, patient 5 experienced a non-vertebral fracture, and patient 10 sustained vertebral fractures, highlighting that significant skeletal fragility may occur even in the absence of densitometric osteoporosis. Notably, four patients (patients 1, 2, 3, and 9) presented with short stature, defined as height below the 3rd percentile according to age and sex based on the Polish growth reference charts [23]. In these cases, height adjustment of BMD results using the HAZ formula led to a noticeable improvement in Z-score values, more accurately reflecting bone status relative to body size, as depicted in Table 3.

Vertebral fractures were the most common manifestation of bone fragility, occurring in 8 patients. Among these, back pain was the leading clinical symptom (Table 1). Only one patient with vertebral fractures remained asymptomatic. Additionally, three children exhibited reduced growth velocity (patients 1, 3, 9). Six patients experienced non-vertebral fractures (patients 1, 3, 6, 11).

### Treatment

Bisphosphonate therapy was administered in nine patients (patients 1, 2, 3, 4, 5, 6, 7, 8, and 11). All of them received intravenous sodium pamidronate at the time of the diagnosis as stated in Table 1, dosed according to internationally accepted clinical protocols [24]. In each case, treatment was supplemented with calcium and vitamin D, with vitamin D administered at age-adjusted doses ranging from 1000 to 2000 IU per day.

Two patients followed alternative treatment strategies. Patient 9, diagnosed with Cushing’s disease, was treated with calcium and vitamin D supplementation alone, as the child’s guardians did not consent to bisphosphonate therapy. Additionally, this patient underwent surgical resection of a pituitary adenoma during the observation period.

Patient 10, carrying a pathogenic *ALPL* gene mutation consistent with hypophosphatasia, received vitamin D supplementation alone, without additional calcium supplementation or bisphosphonate therapy.

Among patients receiving bisphosphonates, patient 6 (with Crohn’s disease) was initially treated with steroids—methylprednisolone and prednisone—which, while controlling the underlying disease, may worsen osteoporosis. Treatment was then switched to infliximab, which effectively maintains disease remission. A detailed summary of therapeutic interventions applied in individual patients is presented in Table 3.

## 4. Discussion

Given the complexities involved in pediatric osteoporosis, a comprehensive and individualized approach to diagnosis and management is essential. Osteoporosis in children is a multifactorial condition that may, in some instances, represent the initial manifestation of an underlying systemic disorder [21]. In our patient cohort, osteoporosis was diagnosed in two individuals (patients 5 and 10) solely on the basis of vertebral fractures. In these patients, the BMD Z-scores in both the TBLH and spine projections were greater than −2.

Non-low energy fractures are a frequent occurrence in childhood, affecting 30–50% of boys and 20–40% of girls before adulthood. The upper limb is the most common fracture site, whereas injuries to the vertebrae or proximal femur are uncommon and together account for less than 2% of all reported fractures in children [25,26,27]. Vertebral fractures, in particular, often serve as the earliest and most sensitive markers of bone fragility in this population. In contrast to peripheral fractures, which generally result from high-energy trauma, vertebral fractures may occur spontaneously or following minimal force, and are frequently underrecognized in the absence of overt clinical signs.

It is noteworthy that in five of our patients (patients 2, 7, 8, 9, and 10), vertebral fractures occurred without concomitant fractures of the long bones. Similar findings were reported by Finnish researchers Mayranpää et al. [28], who studied 1412 children with fractures. Among the 1126 children aged 4–16 years who sustained low-energy or moderate-energy fractures, 1% (11 children) had vertebral fractures. Of these 11 children, five had no history of peripheral fractures; the remaining six had sustained one (*n* = 4), two (*n* = 1), or three (*n* = 1) nonvertebral fractures. Low-energy vertebral fractures were located in the thoracic spine in eight patients, in the cervical spine in one, in the lumbar spine in one, and in both the thoracic and lumbar regions in one patient.

In our study group, the majority of patients (10 out of 11) presented with multilevel vertebral fractures involving both the thoracic and lumbar spine; in one of these patients (patient 3), the cervical spine was also affected. Only one patient (patient 4) had an isolated fracture of a single lumbar vertebra.

Importantly, vertebral fractures are not restricted to primary bone disorders but are observed in children and adolescents with chronic conditions—especially those exposed to long-term glucocorticoid therapy. This highlights the need to consider incorporating lateral spine imaging in the evaluation of at-risk patients, regardless of BMD values. The classification system proposed by Ward (2020) [29] for glucocorticoid-induced secondary osteoporosis offers a valuable framework for assessing the potential for skeletal recovery in various clinical contexts. This stratification, based on the likelihood of spontaneous BMD normalization post-steroid cessation, facilitates personalized treatment planning and supports shared decision-making with patients and families.

In our cohort, three patients had previously received glucocorticoid therapy (two with DMD and one with IBD), and one patient had osteoporosis secondary to endogenous hypercortisolism due to Cushing’s disease. It is noteworthy that growth retardation and short stature accompanying vertebral fractures (osteoporosis) were present not only in both patients with DMD, but also in the remaining two cases in which hypercortisolism—whether exogenous, as in inflammatory bowel disease treated with glucocorticoids, or endogenous, as in Cushing’s disease—was a major factor impairing bone mineral density. Therefore, in pediatric patients presenting with osteoporosis and growth failure, primary and secondary causes of hypercortisolism should be prioritized in the differential diagnosis.

In addition to iatrogenic causes, endogenous hypercortisolism is an important consideration in pediatric osteoporosis. Patient 9 was slim and had Cushing disease secondary to a pituitary adenoma. Approximately 90% of children with Cushing’s disease present with both obesity and growth failure [30], making the absence of obesity in our patient atypical. Notably, osteoporosis was one of the earliest manifestations of the underlying disorder, underscoring the need to consider hypercortisolism in the differential diagnosis of pediatric osteoporosis—even in the absence of classic clinical features.

In children with endogenous Cushing’s, excess cortisol profoundly disrupts bone metabolism through the same mechanisms as exogenous glucocorticoids, but resolution of hypercortisolemia—most often via transsphenoidal surgery for pituitary adenomas—allows gradual normalization of bone turnover, GH/IGF-I, sex steroid, calcium, and PTH homeostasis [31].

It is of particular relevance that vertebral fractures may be present even when routine biochemical markers of calcium-phosphate metabolism are within normal limits. For instance, a recent case report by Shimazaki et al. [32] described an 11-year-old boy with idiopathic juvenile osteoporosis who presented with multiple thoracic and lumbar vertebral compression fractures, yet had completely normal laboratory results, including serum calcium, phosphorus, bone alkaline phosphatase, intact parathyroid hormone, and vitamin D levels. Several cases in the current study exemplify this dissociation, with fractures occurring despite normal serum calcium, phosphate, and parathyroid hormone levels. This observation underscores the limitations of standard laboratory tests in detecting localized or early-stage skeletal fragility and highlights the need for heightened clinical vigilance. On the other hand, Finnish researchers demonstrated that patients with vertebral compression fractures had lower vitamin D levels, reduced dietary calcium intake, and lower lumbar spine BMD Z-scores compared with fracture-prone children without vertebral involvement [28].

Thus, clinicians should maintain a high index of suspicion in the presence of musculoskeletal symptoms or radiographic evidence suggestive of osteoporosis, even in the absence of overt biochemical derangements.

Conversely, in rare genetic disorders such as HPP, skeletal fragility stems from an inherited enzyme deficiency—specifically, insufficient activity of TNSALP, encoded by the *ALPL* gene. This enzyme is essential for hydrolyzing inorganic pyrophosphate (PPi), a potent inhibitor of bone mineralization, into inorganic phosphate (Pi), which is required to form hydroxyapatite crystals in the bone matrix. When TNSALP activity is deficient, PPi accumulates in the extracellular matrix, blocking hydroxyapatite deposition and leading to rickets in children or osteomalacia in adults—conditions marked by skeletal weakness and an elevated risk of fractures and pseudofractures [33]. Osteoporosis is not considered a typical feature of childhood hypophosphatasia, and vertebral fractures are even more rarely reported; While case reports in adults have documented vertebral fractures [34], pediatric fractures typically involve the long bones rather than the spine. This distinction highlights differences in fracture patterns between children and adults with similar underlying bone fragility disorders.

Therefore, the clinical presentation of Patient 10, who exhibited both reduced bone mineral density and compression fractures of the spine, represents an unusual and noteworthy phenotype within the known spectrum of the disease [35]. What is more, conventional anti-resorptive therapies—such as bisphosphonates—are not only ineffective in HPP but may worsen the skeletal pathology. Bisphosphonates can bind to zinc or magnesium cofactors needed by any residual TNSALP, further suppressing mineralization; case series report worsened atypical fractures in adult HPP patients treated with these agents [36]. Thus, in HPP, where the fracture etiology is fundamentally a failure of mineral deposition rather than excessive resorption, anti-resorptives are contraindicated. It is essential to exclude hypophosphatasia before initiating bisphosphonate therapy; therefore, measurement of ALP activity should be included in the calcium–phosphate metabolism panel performed during differential diagnosis. Causal treatment of patients with hypophosphatasia involves enzyme replacement therapy with recombinant asfotase alfa, a mineral-targeted form of TNSALP. Clinical trials confirm it restores mineralization, improves radiographic bone healing, and reduces fracture rates in pediatric and adult HPP [37].

Pathogenic variants in genes such as *COL1A1*, *COL1A2*, *LRP5*, and *SGMS2* have been well-documented as monogenic causes of childhood-onset osteoporosis and vertebral fragility. Mutations in *COL1A1* and *COL1A2*, which encode the pro-α1 and pro-α2 chains of type I collagen, respectively, are the most common cause of OI [38]. Vertebral fractures can be the first manifestation of OI or may indicate disease progression. In the case of patient 11 and 12, the first symptoms were back pain, which was most likely related to vertebral fractures. In a recent study [39], 67 patients with type I OI were included: 70% developed vertebral fractures during follow-up, predominantly in the thoracic spine, and 50% had fractures before the age of 5 years. At diagnosis, approximately 50% of patients already presented with vertebral fractures, typically around age 5.

Mutations in LRP5, a gene encoding a co-receptor in the Wnt/β-catenin signaling pathway crucial for osteoblast differentiation and activity, are associated with osteoporosis-pseudoglioma syndrome (OPPG). This autosomal recessive disorder presents with extremely low bone mass, vertebral compression fractures, and skeletal deformities starting in early childhood, often accompanied by visual impairment due to abnormal retinal vascularization [40]. Heterozygous loss-of-function variants in LRP5 have been associated with primary osteoporosis in children and adolescents, with variable skeletal severity and often no ocular involvement; in patient 5, a heterozygous variant was identified, consistent with this spectrum of LRP5-related primary osteoporosis. Our patient presented with back pain and vertebral fractures at Th12 and L1, in addition to a history of forearm fractures. What is more, no low bone mass was detected in DXA.

In comparison, a patient described by Fahiminiya et al. [41] sustained a total of seven low-energy long-bone fractures involving the radius/ulna, humerus, and tibia/fibula. At first evaluation at age 6, that patient appeared generally healthy, yet exhibited multiple vertebral compression fractures and markedly reduced BMD. Compared with this literature case, our patient shows a milder reduction in bone mineral density and a less extensive fracture history, despite similar vertebral involvement. Similarly, Stürznickel et al. [42] reported fractures in 74% of their cohort, with vertebral fractures in 36% and peripheral fractures in 58% of patients, and consistently low bone formation across all individuals; however, only 62% had BMD Z-scores ≤ −2.0, highlighting that fracture susceptibility may not always correlate directly with DXA measurements. These observations suggest that LRP5-associated pediatric osteoporosis exhibits significant phenotypic variability, and that fracture risk may not directly correlate with DXA measurements alone, highlighting the need for individualized clinical assessment.

More recently, loss-of-function mutations in *SGMS2*, which encodes sphingomyelin synthase 2, have been implicated in Autosomal Dominant Osteoporosis with Calvarial Doughnut Lesions (ADOCDL). Affected individuals show generalized low bone mineral density, frequent vertebral fractures, and abnormal skull ossification patterns. Patient 7 and Patient 8 in our study, whom were siblings, both harboring *SGMS2* mutations, demonstrated the more classical ADOCDL phenotype, including palpable sclerotic protuberances of the skull, consistent with calvarial bone overgrowth, along with radiologically confirmed vertebral fractures. Although the precise mechanism remains unclear, studies suggest that *SGMS2* may influence bone mineralization via effects on plasma membrane–bound sphingomyelin metabolism [16]. Importantly, vertebral compression fractures have been reported as an early and prominent manifestation in several families with SGMS2-related osteoporosis, underscoring its clinical significance in pediatric bone fragility diagnostics [43]. It should be emphasized that the literature contains limited data regarding treatment, although individual reports have noted beneficial effects of bisphosphonates [16]. In our patient cohort, bisphosphonate therapy was implemented for this reason.

Collectively, these genetic findings emphasize the need for targeted molecular diagnostics in children presenting with unexplained vertebral fractures, especially in the absence of secondary causes or with a positive family history.

Pediatric IBD is associated with reduced bone mineral density and significant disturbances in the RANKL/OPG axis, particularly in Crohn’s disease [44]. The therapeutic response in chronic inflammatory conditions such as IBD is influenced not only by bone-directed treatments but also by effective control of systemic inflammation. Importantly, IBD is often initially managed with glucocorticoid therapy, which exerts deleterious effects on bone metabolism by inhibiting osteoblast function, promoting osteoclast-mediated resorption, and impairing calcium balance—contributing to reductions in BMD and increasing the risk of osteoporosis [45]. In our patient, DXA scanning was not performed at the time of initial diagnosis of IBD nor during the first years of treatment, which is currently standard practice at the authors’ center. Consequently, vertebral fracture and the associated pain, which limited her mobility, represented the first manifestation of osteoporosis.

A study of over 737 cases of Crohn’s disease found that young children (<12 years) were at higher risk and had a higher prevalence of fractures compared to non-IBD controls [28]. In a systematic review and meta-analysis of adult patients with IBD, individuals with IBD were found to have a 38% greater overall fracture risk and a significantly higher risk of vertebral fractures compared to healthy controls [46]. Vertebral fracture risk in adults has been reported at 22% [47]. Data on vertebral fractures in children are limited [48]; one cross-sectional study of 216 children with very early-onset IBD (onset before age 6) reported vertebral fractures in 1.4% of the cohort [44,49].

Similarly, neuromuscular conditions such as DMD exemplify a multifactorial clinical scenario in which bone fragility arises from the synergistic effects of chronic pharmacotherapy, progressive muscle weakness, and subsequent mechanical unloading. In DMD, long-term glucocorticoid therapy—typically administered in continuous daily regimens—remains the standard of care to preserve motor strength and prolong ambulation, yet it concurrently induces reductions in BMD, delays in linear growth, and suppression of endogenous bone turnover [50]. In our study, we presented two patients with DMD, both of whom had vertebral fractures accompanied by growth retardation and short stature. Cross-sectional analyses have demonstrated that longer duration and higher daily doses of systemic glucocorticoids correlate with lower lumbar BMD Z-scores, shorter stature, greater delay of bone age, and an increased odds of vertebral fractures [50,51]. Reduced mobility dramatically elevates fracture risk: up to 60% suffer low-trauma long-bone fractures by mid-adolescence, and vertebral fractures occur in 30–60% of patients, often asymptomatically, causing chronic back pain, vertebral deformity, and accelerated functional decline [52]. In the study by Liaw et al. [53] involving 155 boys with DMD, the first fracture was vertebral in 55% of cases; 41% had non-vertebral fractures, and 4% experienced both. Vertebral fractures occurred in significantly older boys and were associated with longer cumulative exposure to glucocorticoids compared with non-vertebral fractures. Current guidelines advocate regular spine imaging—even in the absence of symptoms—to identify early vertebral fractures [51], alongside periodic assessment of BMD and growth parameters.

Bisphosphonates remain a cornerstone of pharmacological intervention in pediatric osteoporosis, particularly in the presence of vertebral fractures. These agents have demonstrated efficacy in enhancing BMD and promoting vertebral remodeling, which contributes to improved vertebral morphology and structural integrity [54]. Nonetheless, therapeutic decisions should be made judiciously, considering potential contraindications and disease-specific factors. In our study cohort, nearly all patients (with the exception of patients 9 and 10) received bisphosphonate therapy. The parents of patient 9 declined treatment, whereas in the case of the patient with hypophosphatasia, bisphosphonate therapy was contraindicated.

A key limitation of the present study is the relatively small number of patients included. This reflects the rarity of pediatric osteoporosis and vertebral compression fractures, which are uncommon clinical entities in children and adolescents. Consequently, large-scale studies are challenging to conduct, and most evidence in this area is derived from single-center cohorts or case series. Additionally, variability in the timing of diagnosis and treatment initiation could have influenced individual outcomes. Despite these limitations, the findings provide valuable insights into the clinical presentation, management, and long-term implications of pediatric osteoporosis, highlighting areas for future research and the importance of multicenter collaboration to better characterize this rare condition.

## 5. Conclusions

This case series underscores the complexity and heterogeneity of pediatric osteoporosis, a rare yet clinically significant condition that demands heightened awareness among healthcare professionals. Through the analysis of diverse clinical contexts, it highlights the multifactorial nature of pediatric bone fragility, which may arise from primary skeletal disorders or occur secondarily in the context of chronic illnesses, hormonal disturbances, inflammatory conditions, or prolonged glucocorticoid therapy. Importantly, this work draws attention to the often-underrecognized clinical presentation of osteoporosis in children and adolescents, emphasizing that vertebral fractures—frequently asymptomatic or expressed through non-specific pain symptoms such as persistent back pain—can serve as the earliest and sometimes sole manifestation of the disease across a range of underlying diagnoses.

Beyond its immediate clinical manifestations, pediatric osteoporosis may have profound and lasting consequences for skeletal health. Childhood and adolescence constitute critical periods for bone modeling and remodeling, during which the majority of peak bone mass is accrued. Disruption of these processes can result in inadequate bone mineral acquisition, leading to a suboptimal peak bone mass that persists into adulthood and increases lifelong fracture risk. Independently, compromised bone quality and altered skeletal metabolism may adversely affect longitudinal growth, as factors commonly associated with pediatric osteoporosis—including chronic disease, hormonal disturbances, and reduced mechanical loading—can impair normal growth plate function and skeletal development, thereby potentially limiting final adult height.

Collectively, these findings reinforce the importance of vigilant clinical assessment, particularly in high-risk populations, and support a comprehensive, multidisciplinary diagnostic approach that integrates clinical history, fracture assessment, growth patterns, and pubertal development. Ultimately, early recognition and individualized management are essential to prevent long-term skeletal complications and to optimize bone health outcomes across the lifespan of affected children and adolescents.

## Figures and Tables

**Table 1:** Summary of Clinical Data from Pediatric Patients with Osteoporosis and Vertebral Compression Fractures Hospitalized at PMMH-RI, Lodz (2020–2024).

Patient No.	Sex	Age	Tanner Stage	Height [cm]	Height Standard Deviation Score [hSDS]	Short Stature	Weight [kg]	BMI and Mass Percentile Range	Preliminary Symptoms	Background Diagnosis
1	M	9	I	121.0	−2.78	Yes	33.5	22.9 (90–97)	Thoracic pain, growth retardation	Duchenne Muscular Dystrophy (steroid treatment)
2	M	13	II	135.0	−3.86	Yes	30.0	16.5 (10–25)	Back pain, growth retardation	Duchenne Muscular Dystrophy (steroid treatment)
3	F	13	IV	147.0	−2.33	Yes	37.0	17.0 (10–25)	Immobility, overall pain, growth retardation	Crohn’s disease (steroid treatment)
4	F	5	I	109.0	−0.33	No	21.8	18.4 (90–97)	Blue sclera	*COL1A1* gene mutation
5	M	11	II	150.3	0.35	No	48.5	21.5 (75–90)	Back pain	*LRP5* gene mutation
6	M	12	II	153.0	−0.55	No	70.0	29.9 (>97)	Back pain	*COL1A2* gene mutation
7	F	6	I	114.0	−1.26	No	18.9	14.5 (25–50)	Palpable head sclerotic protuberance	*SGMS2* gene mutation
8	F	9	II	128.5	−1.36	No	20.0	12.1 (<3)	Palpable head sclerotic protuberance	*SGMS2* gene mutation
9	M	16	IV	162.4	−2.43	Yes	48.4	18.4 (10–25)	Back pain, growth retardation	Cushing’s disease (pituitary adenoma)
10	F	13	III	160.0	0.10	No	74.0	28.9 (>97)	Back pain	*ALPL* gene mutation
11	M	10	I	136.5	−1.28	No	23.8	12.8 (<3)	Back pain, facial dysmorphia	*COL1A2* gene mutation

**Table 2:** Biochemical Parameters of Pediatric Patients with Osteoporosis and Vertebral Compression Fractures Hospitalized at PMMH-RI, Lodz (2020–2024).

Patient No.	25(OH)D [ng/mL]	Serum Calcium Levels [mmol/L]	Serum Phosphate Levels [mmol/L]	Alkaline Phopshate Activity [U/L]	Parathormone [pg/mL]	Osteocalcin [ng/mL]	Calcium/Creatinine Ratio in the First Urine Portion [mg/mg] N < 0.21 mg/mg
1	38.9	2.48 (2.2–2.7)	1.63 (1.28–1.98)	41 (160–381)	28.9 (15–65)	17.8 (21–108)	0.07
2	37.5	2.29 (2.29–2.66)	1.34 (0.97–1.74)	39 (116–483)	16.4 (15–65)	8.5 (19–159)	0.12
3	38.2	2.40 (2.29–2.66)	1.75 (0.97–1.81)	119 (62–209)	26.3 (15–65)	74.8 (15–151)	0.09
4	30.0	2.55 (2.19–2.51)	1.76 (0.81–1.94)	190 (156–386)	27.2 (15–65)	100.8 (16–152)	0.09
5	51.5	2.46 (2.29–2.66)	1.38 (0.97–1.74)	292 (116–483)	19.8 (15–65)	113.4 (19–159)	0.38
6	43.0	2.51 (2.19–2.64)	1.45 (0.97–1.94)	325 (178–455)	22.2 (15–65)	70.3 (19–159)	0.09
7	21.7	2.25 (2.22–2.51)	1.48 (0.81–1.94)	267 (116–515)	25.9 (15–65)	41.0 (16–152)	0.07
8	50.0	2.35 (2.19–2.51)	1.61 (0.81–1.94)	234 (156–386)	27.9 (15–65)	75.4 (16–152)	0.09
9	46.7	2.41 (2.22–2.66)	1.30 (0.97–1.74)	107 (58–237)	22.9 (15–65)	23.7 (12–114)	0.33
10	26.7	2.26 (2.29–2.66)	1.65 (0.97–1.81)	60 (62–209)	35.5 (15–65)	58.0 (15–151)	0.08
11	27.4	2.40 (2.22–2.51)	1.76 (0.97–1.94)	142 (120–488)	15.1 (15–65)	84.8 (19–159)	0.30

**Table 3:** Bone Density Measurements, Fracture Assessment, and Treatment Details in Pediatric Patients with Osteoporosis and Vertebral Compression Fractures Hospitalized at PMMH-RI, Lodz (2020–2024).

Patient No.	DXA Z-Score Spine with Height-Adjustment in Case of Short Stature	DXA Z-Score TBLH with Height-Adjustment in Case of Short Stature	Identified Vertebrae Fractures	Other Identified Fractures	Implemented Treatment
1	−1.70 HAZ = −0.72	−3.70 HAZ = −2.16	Th7, Th11, L1	Forearm	bisphosphonate therapy + calcium, vitamin D
2	−3.90 HAZ = −1.46	−8.00 HAZ = −5.65	Th5-Th12, L1-L4	-	bisphosphonate therapy + calcium, vitamin D
3	−3.20 HAZ = −2.25	−4.20 HAZ = −3.20	C7, Th5, Th7-Th12, L1, L3, L4	Sternum	bisphosphonate therapy + calcium, vitamin D
4	−2.60	−1.20	L1	Tibia	bisphosphonate therapy + calcium, vitamin D
5	−1.30	−0.70	Th12, L1	Forearm (2013), (2017)	bisphosphonate therapy + calcium, vitamin D
6	−3.50	−4.90	Th12, L1	Femur, shoulder	bisphosphonate therapy + calcium, vitamin D
7	−3.10	−2.30	Multilevel vertebral fractures (thoracic and lumbar vertebrae)	-	bisphosphonate therapy + calcium, vitamin D
8	−2.70	−3.30	Th5-Th9, L4, L5	-	bisphosphonate therapy + calcium, vitamin D
9	−5.50 HAZ = −4.60	−4.50 HAZ = −3.49	Th9, Th11, L1	-	endoscopic transsphenoidal surgery of pituitary adenoma; parental refusal for introducing bisphosphonate treatment; calcium + vitamin D
10	−1.80	−1.00	Th11-L3	-	vitamin D
11	−4.30	−3.50	Th11-L5	Humerus	bisphosphonate therapy + calcium, vitamin D

## Data Availability

The datasets used and/or analyzed within the framework of this study are available from the corresponding author on reasonable request.
